# Exosomes in Colorectal Cancer: From Tumor Biology to Diagnostic and Therapeutic Applications

**DOI:** 10.3390/medicina62081538

**Published:** 2026-08-11

**Authors:** Ugur Topal, Cihan Zamur

**Affiliations:** 1 Department of Surgical Oncology, Cukurova University, Adana 01330, Turkey; 2 Department of Pediatric, Sehitkamil State Hospital, Gaziantep 27500, Turkey; cihanzamur@gmail.com

**Keywords:** colorectal cancer, exosomes, extracellular vesicles, metastasis, liquid biopsy, therapeutic resistance

## Abstract

Colorectal cancer (CRC) remains a leading cause of cancer-related morbidity and mortality worldwide despite advances in screening, surgical techniques, and systemic therapies. In recent years, exosomes—nanoscale extracellular vesicles involved in intercellular communication—have emerged as critical regulators of CRC biology. Exosomes mediate tumor progression, metastatic dissemination, immune evasion, and therapeutic resistance through the transfer of bioactive molecules including proteins, lipids, and non-coding RNAs. Moreover, exosomes have gained increasing attention as promising liquid biopsy tools and therapeutic platforms due to their stability, biocompatibility, and ability to reflect tumor molecular dynamics. This review provides a comprehensive overview of exosome biogenesis, molecular composition, and their functional roles in CRC pathogenesis. We further discuss their diagnostic and prognostic value, involvement in therapeutic resistance, and emerging therapeutic applications, with particular emphasis on translational and surgical oncology implications. Finally, we highlight current limitations and future perspectives for clinical integration of exosome-based strategies in CRC management.

## 1. Introduction

Colorectal cancer (CRC) continues to represent a major global health challenge, ranking among the most frequently diagnosed malignancies and a leading cause of cancer-related mortality worldwide [1,2]. Despite substantial progress in screening strategies, surgical techniques, and systemic therapies, disease recurrence and metastatic progression remain the principal determinants of long-term survival. In particular, hepatic and peritoneal dissemination characterize advanced-stage CRC and are associated with limited therapeutic efficacy and unfavorable prognosis, underscoring the need for a deeper mechanistic understanding of tumor progression and novel therapeutic paradigms [3].

In recent years, the conceptual framework of cancer biology has expanded beyond tumor-cell-intrinsic alterations to encompass the dynamic interplay between malignant cells and the tumor microenvironment (TME). Within this context, extracellular vesicles (EVs), and more specifically exosomes, have emerged as critical mediators of intercellular communication [4,5]. Exosomes are nanoscale (30–150 nm) lipid bilayer-enclosed vesicles originating from the endosomal compartment through the inward budding of multivesicular bodies (MVBs) and their subsequent fusion with the plasma membrane [6]. Their molecular cargo—comprising proteins, lipids, genomic DNA fragments, messenger RNAs, and a diverse repertoire of non-coding RNAs, including microRNAs (miRNAs) and long non-coding RNAs (lncRNAs)—enables the horizontal transfer of bioactive molecules, thereby reprogramming recipient cells and modulating both local and systemic tumor-promoting processes [5,7].

Accumulating evidence implicates exosomes as central regulators in multiple hallmarks of colorectal carcinogenesis. Tumor-derived exosomes actively contribute to cellular proliferation, invasion, and epithelial–mesenchymal transition (EMT), while also orchestrating the formation of pre-metastatic niches, particularly within the hepatic microenvironment [8,9]. Moreover, exosome-mediated crosstalk plays a pivotal role in immune modulation, facilitating tumor immune evasion through mechanisms such as T-cell suppression, macrophage polarization, and the dissemination of immune checkpoint molecules, including programmed death-ligand 1 (PD-L1) [10].

Beyond their functional role in tumor biology, exosomes have garnered significant attention as minimally invasive biomarkers. Their relative stability in biological fluids, coupled with their capacity to reflect the molecular landscape of their cell of origin, positions exosomes as promising candidates for liquid biopsy applications in CRC, including early detection, prognostication, and real-time monitoring of therapeutic response [11,12]. Concurrently, their intrinsic biocompatibility and low immunogenicity have prompted investigation into their utility as novel therapeutic platforms, particularly in the context of targeted drug delivery, RNA-based therapies, and cancer immunotherapy [13].

Nevertheless, the clinical translation of exosome-based strategies remains constrained by several critical challenges, including the lack of standardized isolation and characterization methodologies, the inherent heterogeneity of exosomal populations, and the incomplete delineation of their context-dependent biological functions [4,6].

In this review, we provide a comprehensive and critical appraisal of the current evidence regarding the role of exosomes in colorectal cancer. We focus on their mechanistic contributions to tumor progression, metastatic dissemination, immune regulation, and therapeutic resistance, while also exploring their emerging diagnostic and therapeutic applications. Particular emphasis is placed on the translational and surgical oncology implications of exosome biology, aiming to bridge the gap between experimental findings and clinical practice.

Although exosome biology has been extensively investigated from a molecular perspective, its potential applications in surgical oncology remain incompletely explored. In colorectal cancer, perioperative management increasingly relies on precision biomarkers capable of identifying minimal residual disease (MRD), predicting recurrence following curative-intent surgery, monitoring metastatic progression, and supporting individualized treatment decisions. This review therefore places particular emphasis on the translational relevance of exosome-based biomarkers within surgical oncology, discussing their potential integration into postoperative surveillance strategies and future precision medicine approaches.

Although several excellent reviews have summarized the biological functions of exosomes in colorectal cancer, most have primarily focused on molecular mechanisms or experimental findings. Comparatively less attention has been given to the clinical translation of these discoveries, particularly from the perspective of surgical oncology. This review therefore aims to bridge this gap by critically integrating recent evidence on exosome biology with current challenges in biomarker validation, therapeutic resistance, perioperative surveillance, postoperative recurrence monitoring, and future clinical implementation. Particular emphasis is placed on identifying unresolved methodological issues and translational barriers that must be addressed before exosome-based technologies can become part of routine colorectal cancer management.

## 2. Methodology

This review was conducted as a narrative review based on a structured literature search to summarize current evidence regarding the biological and clinical roles of exosomes in colorectal cancer.

A comprehensive literature search was performed using the PubMed/MEDLINE, Scopus, and Web of Science databases. The search primarily included studies published between January 2015 and May 2026, while seminal earlier publications describing exosome biology and extracellular vesicles were also included when considered essential for providing scientific background. During the preparation of this manuscript, we used OpenAI ChatGPT (GPT-5.5; OpenAI, San Francisco, CA, USA) and Google NotebookLM (web version; Google LLC, Mountain View, CA, USA) to assist with language refinement, text organization, and the conceptual planning of original figures. All generated content was critically reviewed, verified, and edited by the authors. The authors take full responsibility for the accuracy, interpretation, and content of this publication.

The following search terms and combinations were used:

- “colorectal cancer” AND exosomes
- “extracellular vesicles” AND colorectal cancer
- exosomes AND metastasis
- exosomes AND liquid biopsy
- exosomes AND therapeutic resistance
- exosomes AND immunotherapy
- exosomes AND biomarker
- exosomes AND drug delivery

Priority was given to peer-reviewed original research articles, systematic reviews, meta-analyses, and high-impact review articles published in English. The literature selection focused on studies investigating:

exosome biogenesis and molecular composition,

mechanisms of tumor progression and metastasis,

diagnostic and prognostic biomarkers,

therapeutic resistance,

and emerging therapeutic applications in colorectal cancer.

Studies unrelated to colorectal cancer, conference abstracts without full manuscripts, duplicate publications, editorials, commentaries, and articles lacking sufficient scientific data were excluded.

The retrieved publications were evaluated by the authors according to their scientific relevance, methodological quality, novelty, and contribution to the current understanding of exosome biology in colorectal cancer. The selected studies were synthesized narratively to provide a comprehensive overview of the field.

### 2.1. Biogenesis and Molecular Composition of Exosomes

Exosomes constitute a specialized subset of extracellular vesicles (EVs) originating from the endosomal trafficking pathway, distinguished by their biogenesis, molecular composition, and functional properties [4,14]. Their formation is a tightly regulated, multistep process involving endosomal maturation, cargo sorting, and vesicular secretion, which collectively determine their biological activity and target specificity.

### 2.2. Biogenesis of Exosomes

The biogenesis of exosomes is initiated by the inward budding of the limiting membrane of early endosomes, leading to the formation of intraluminal vesicles (ILVs) within multivesicular bodies (MVBs) [6]. These MVBs subsequently undergo one of two fates: fusion with lysosomes for degradation or trafficking toward the plasma membrane, where they fuse and release ILVs into the extracellular space as exosomes [6,15].

This process is primarily regulated by the endosomal sorting complex required for transport (ESCRT) machinery, a highly conserved system composed of four multiprotein complexes (ESCRT-0, -I, -II, and -III) and associated accessory proteins such as ALIX and TSG101 [16]. ESCRT-dependent sorting facilitates the selective incorporation of ubiquitinated cargo into ILVs, thereby influencing the molecular profile of secreted exosomes.

In parallel, ESCRT-independent mechanisms have also been described, involving lipid-raft-associated pathways, ceramide formation mediated by neutral sphingomyelinase (nSMase), and tetraspanin-enriched microdomains (e.g., CD9, CD63, CD81) [17]. These alternative pathways highlight the complexity and redundancy of exosome biogenesis, particularly in cancer cells, where dysregulated signaling pathways may alter vesicle production.

The secretion of exosomes is further regulated by members of the Rab GTPase family, including Rab27a, Rab27b, Rab11, and Rab35, which coordinate vesicular trafficking, docking, and membrane fusion [18]. In colorectal cancer, aberrant expression of these regulators has been associated with increased exosome release and enhanced metastatic potential.

### 2.3. Molecular Composition of Exosomes

The molecular cargo of exosomes reflects both their endosomal origin and the physiological or pathological state of the parental cell. Exosomes are enriched in a distinct repertoire of biomolecules, broadly categorized into proteins, lipids, and nucleic acids [14,19].

### 2.4. Protein Cargo

Exosomal proteins include:

- Membrane transport and fusion proteins (e.g., annexins, flotillins);
- Tetraspanins (CD9,CD63,CD81), commonly used as exosomal markers;
- ESCRT-related proteins (TSG101, ALIX);
- Heat shock proteins (HSP70, HSP90);
- Tumor-associated proteins (e.g., mutant KRAS, EGFR in CRC).

These proteins not only serve as structural components but also mediate target cell recognition, uptake, and downstream signaling [20].

### 2.5. Lipid Composition

Exosomes are enriched in specific lipids such as cholesterol, sphingomyelin, ceramide, and phosphatidylserine, contributing to membrane rigidity, stability, and fusion capacity [17]. These lipid components are not merely structural but actively participate in vesicle formation and intercellular signaling.

### 2.6. Nucleic Acid Cargo

A defining feature of exosomes is their ability to transport nucleic acids, including:

- Messenger RNAs (mRNAs);
- MicroRNAs (miRNAs);
- Long non-coding RNAs (lncRNAs);
- Circular RNAs (circRNAs);
- Double-stranded DNA fragments.

In colorectal cancer, exosomal miRNAs such as miR-21, miR-92a, and miR-1246 have been implicated in tumor progression, metastasis, and therapeutic resistance [19,21]. Importantly, these nucleic acids are protected from degradation within the lipid bilayer, enabling their stable circulation in biological fluids and functional delivery to recipient cells.

### 2.7. Functional Implications in Colorectal Cancer

The selective packaging of bioactive molecules into exosomes is not a passive process but rather a highly regulated event that dictates their functional impact on recipient cells. In CRC, tumor-derived exosomes can reprogram stromal fibroblasts, endothelial cells, and immune cells, thereby reshaping the tumor microenvironment to favor tumor growth and dissemination [9].

Moreover, exosomal cargo is dynamically modulated under conditions such as hypoxia, chemotherapy exposure, and oncogenic signaling activation, suggesting that exosomes serve as adaptive mediators of tumor evolution and treatment resistance [22].

### 2.8. Exosome Isolation and Standardization

The clinical translation of exosome-based biomarkers is currently limited by methodological heterogeneity in extracellular vesicle isolation and characterization. Several isolation techniques are available, including differential ultracentrifugation, size-exclusion chromatography (SEC), polymer-based precipitation, immunoaffinity capture, and microfluidic approaches. Differential ultracentrifugation remains the most widely used research method but is time-consuming and susceptible to contamination with non-vesicular particles. SEC provides higher purity while preserving vesicle integrity, whereas precipitation methods are simple but often co-isolate proteins and lipoproteins. Emerging microfluidic technologies offer rapid processing and require smaller sample volumes but still require large-scale validation.

Reliable characterization is equally important and generally requires complementary techniques such as nanoparticle tracking analysis, electron microscopy, and detection of canonical EV markers including CD9, CD63, CD81, TSG101, and ALIX. Variability in pre-analytical handling, isolation protocols, and analytical methods contributes substantially to the inconsistent performance of proposed exosomal biomarkers across studies.

To improve reproducibility, the International Society for Extracellular Vesicles (ISEV) published the MISEV2018 guidelines, recommending standardized reporting of sample collection, isolation procedures, vesicle characterization, and functional validation. Broader implementation of these recommendations will be essential for multicenter biomarker validation and future clinical translation of exosome-based technologies in colorectal cancer.

### 2.9. Role of Exosomes in Colorectal Cancer Progression and Metastasis

**Figure 1:**
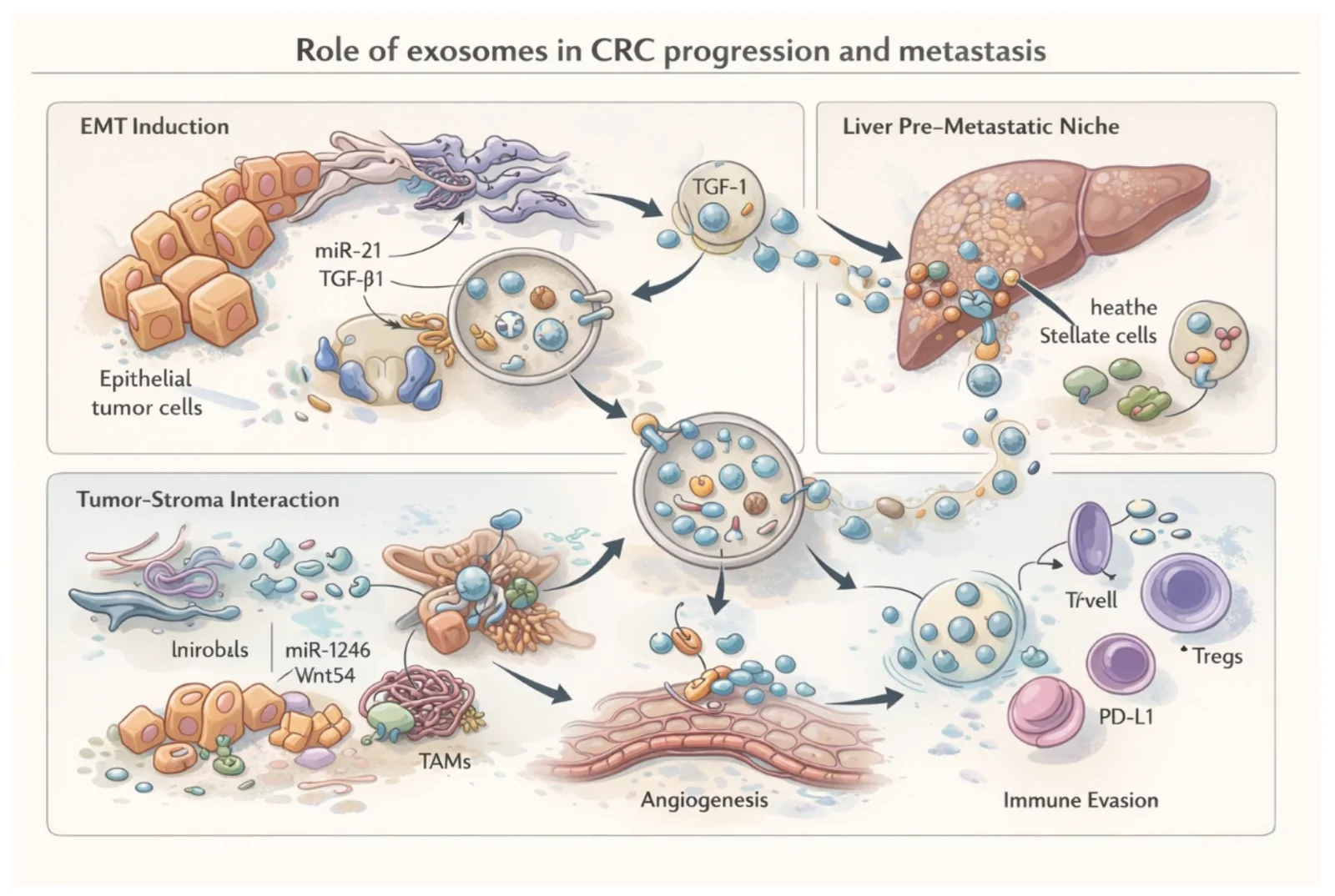
Role of exosomes in CRC progression and metastasis. Depiction of EMT induction, liver pre-metastatic niche formation, angiogenesis, immune evasion, and tumor-stroma interactions.

Exosomes have emerged as central mediators of tumor progression in colorectal cancer (CRC), orchestrating a complex network of intercellular communication that extends beyond the primary tumor site. Through the horizontal transfer of oncogenic proteins, nucleic acids, and lipids, tumor-derived exosomes actively modulate both local tumor microenvironment (TME) dynamics and systemic processes that facilitate metastatic dissemination [4,9]. Exosome-mediated mechanisms of tumor progression and metastasis are summarized in Figure 1.

## 3. CRC-Specific Exosomal Mechanisms

In colorectal cancer, exosome-mediated communication is shaped by the unique biology of the intestinal tumor microenvironment. CRC-derived exosomes interact with intestinal epithelial cells, cancer-associated fibroblasts, endothelial cells, macrophages, neutrophils, and T cells, thereby promoting a pro-tumorigenic niche characterized by inflammation, immune suppression, extracellular matrix remodeling, and angiogenesis. These vesicles can transfer CRC-associated molecular cargo, including miR-21, miR-92a, miR-1246, miR-25-3p, miR-320d, mutant KRAS, and PD-L1, which are involved in tumor invasion, metastatic dissemination, immune escape, and treatment resistance.

A particularly clinically relevant aspect of CRC exosome biology is liver metastasis. CRC-derived exosomes can prime the hepatic microenvironment before the arrival of tumor cells by activating Kupffer cells, hepatic stellate cells, and endothelial cells. This process promotes inflammatory signaling, vascular permeability, extracellular matrix remodeling, and formation of a permissive pre-metastatic niche. Exosomal molecules such as miR-1246, miR-25-3p, and specific integrin profiles have been implicated in hepatic niche formation and organotropic metastasis, supporting their potential use as biomarkers for early metastatic risk stratification.

CRC-associated exosomes also contribute to immune modulation within both the primary intestinal tumor microenvironment and distant metastatic sites. Tumor-derived exosomal PD-L1 may suppress cytotoxic T-cell activity, while other exosomal signals can promote regulatory T-cell expansion and macrophage polarization toward an immunosuppressive M2 phenotype. These mechanisms may partly explain immune escape in microsatellite-stable CRC and could influence response to immune checkpoint inhibitors, although robust clinical validation is still lacking.

From a therapeutic standpoint, CRC-derived exosomes are increasingly linked to resistance against 5-fluorouracil, oxaliplatin, anti-EGFR therapy, and immunotherapy. Exosomal miR-21, miR-130b-3p, miR-454-3p, mutant KRAS, HER2/MET-related signals, and PD-L1 represent clinically relevant candidates for resistance monitoring. However, most of these findings remain based on preclinical or retrospective studies, and prospective validation is needed before exosomal biomarkers can be integrated into routine CRC management.

### 3.1. Exosomes and Tumor Growth, Invasion, and EMT

A critical hallmark of CRC progression is the acquisition of invasive and migratory capabilities, frequently driven by epithelial–mesenchymal transition (EMT). Exosomes derived from CRC cells have been shown to induce EMT in recipient epithelial cells by transferring specific microRNAs, transcription factors, and signaling molecules that regulate key pathways such as Wnt/β-catenin, TGF-β, and PI3K/AKT [23,24].

Among these, exosomal miR-21 and miR-200 family members have been extensively implicated in EMT induction, promoting downregulation of E-cadherin and upregulation of mesenchymal markers such as N-cadherin and vimentin [25]. Furthermore, exosomal cargo can activate stromal fibroblasts into cancer-associated fibroblasts (CAFs), which in turn secrete pro-tumorigenic factors that reinforce tumor growth and invasion [26].

### 3.2. Pre-Metastatic Niche Formation and Organotropism

One of the most compelling roles of exosomes in CRC is their involvement in the establishment of pre-metastatic niches. Tumor-derived exosomes can “educate” distant organ microenvironments prior to the arrival of circulating tumor cells, thereby creating a permissive niche for metastatic colonization [27].

In colorectal cancer, the liver represents the predominant site of metastasis, and exosomes play a pivotal role in hepatic niche priming. Mechanistically, exosomal integrins have been shown to determine organ-specific metastasis (organotropism), with distinct integrin expression patterns directing vesicles to specific target tissues [27]. Once internalized by resident liver cells—such as Kupffer cells and hepatic stellate cells—exosomes induce pro-inflammatory and fibrotic responses, promoting extracellular matrix remodeling and vascular permeability [7].

Additionally, exosomal miRNAs such as miR-25-3p have been demonstrated to enhance vascular permeability and angiogenesis by targeting endothelial tight junction proteins, thereby facilitating metastatic dissemination [28].

### 3.3. Angiogenesis and Vascular Remodeling

Exosomes contribute to tumor angiogenesis by transferring pro-angiogenic factors and regulatory RNAs to endothelial cells. CRC-derived exosomes can activate VEGF signaling pathways and modulate endothelial cell proliferation, migration, and tube formation [29].

Moreover, hypoxia—a common feature of the tumor microenvironment—has been shown to alter exosomal cargo composition, enriching vesicles with angiogenesis-promoting miRNAs and proteins. These hypoxia-induced exosomes further amplify neovascularization, thereby supporting tumor expansion and metastatic spread [30].

### 3.4. Immune Modulation and Tumor Immune Escape

Exosome-mediated immune modulation represents a critical mechanism by which CRC evades immune surveillance. Tumor-derived exosomes can suppress anti-tumor immune responses through multiple pathways, including inhibition of cytotoxic T lymphocytes, expansion of regulatory T cells (Tregs), and polarization of macrophages toward an immunosuppressive M2 phenotype [31].

A particularly important mechanism involves the transfer of exosomal PD-L1, which can directly inhibit T-cell activation and function, contributing to systemic immune suppression [10]. This exosome-mediated checkpoint signaling may also influence response to immunotherapy, representing a potential biomarker and therapeutic target.

### 3.5. Interaction with the Tumor Microenvironment

Exosomes function as key regulators of bidirectional communication between tumor cells and the surrounding microenvironment. In CRC, this includes interactions with CAFs, endothelial cells, immune cells, and extracellular matrix components. Through these interactions, exosomes promote a pro-tumorigenic niche characterized by inflammation, immunosuppression, and enhanced invasiveness [4,26].

Importantly, exosomal signaling is highly dynamic and context-dependent, with cargo composition influenced by oncogenic mutations (e.g., KRAS), metabolic status, and therapeutic pressures. This adaptability underscores the role of exosomes as drivers of tumor heterogeneity and disease progression.

### 3.6. Diagnostic and Prognostic Value of Exosomes in Colorectal Cancer

Exosomes have gained substantial attention as minimally invasive biomarkers in colorectal cancer (CRC), owing to their stability in biological fluids and their capacity to reflect the molecular landscape of their parental tumor cells. Recent advances (particularly between 2023 and 2025) have further strengthened their potential role in early detection, prognostication, and therapeutic monitoring, positioning exosomes at the forefront of liquid biopsy technologies [32,33].

### 3.7. Exosomes as Liquid Biopsy Tools

Liquid biopsy has emerged as a transformative approach in oncology, offering real-time, non-invasive access to tumor-derived molecular information. In this context, exosomes provide several advantages over circulating tumor DNA (ctDNA) and circulating tumor cells (CTCs), including enhanced stability of their cargo and protection from enzymatic degradation due to their lipid bilayer structure [4].

Recent studies have demonstrated that exosomal microRNAs (miRNAs) exhibit high diagnostic accuracy in CRC. For instance, plasma exosomal miR-99b-5p, either alone or in combination with other miRNAs, has shown promising performance in CRC detection, suggesting its utility in screening strategies [34]. Similarly, emerging panels of exosomal miRNAs have demonstrated improved sensitivity and specificity compared to single biomarkers, highlighting the importance of multi-marker approaches [35].

A 2024 systematic review further confirmed that circulating and exosomal miRNAs represent robust candidates for early CRC detection, particularly in early-onset disease, where traditional screening methods may be less effective [33].

### 3.8. Emerging Exosomal RNA Biomarkers (2024–2025 Advances)

Beyond classical miRNAs, recent studies have expanded the spectrum of exosomal biomarkers to include circular RNAs (circRNAs) and other non-coding RNAs.

A notable 2021 study demonstrated that exosomal circSCP2 promotes CRC progression and metastasis through interaction with RNA-binding proteins and miRNA sponging mechanisms. Importantly, elevated levels of circSCP2 in serum exosomes were significantly associated with advanced tumor stage, suggesting its prognostic relevance [36].

Similarly, exosomal miR-205-5p has recently been identified as a potential diagnostic biomarker, with elevated levels observed in CRC patients compared to healthy controls, further supporting the expanding repertoire of clinically relevant exosomal RNAs [37]. These findings underscore a paradigm shift from single biomarker approaches toward multi-omics exosomal profiling, integrating miRNA, circRNA, and protein signatures [35,36].

### 3.9. Prognostic Value and Metastasis Prediction

Exosomes are increasingly recognized as predictors of disease progression and metastatic potential. Their cargo composition reflects tumor aggressiveness and dynamic tumor evolution. A particularly impactful 2025 study demonstrated that tumor-derived exosomal miR-1246 plays a direct role in promoting liver metastasis by activating hepatic stellate cells, thereby facilitating the formation of a pro-metastatic microenvironment [21]. Given the clinical significance of liver metastases in CRC, such findings highlight the potential of exosomal biomarkers in early metastasis prediction and risk stratification. In parallel, exosomal miRNAs involved in angiogenesis and metastatic signaling—such as miR-320d—have been shown to activate key oncogenic pathways (e.g., JAK/STAT), further linking exosomal cargo to aggressive tumor phenotypes [38].

### 3.10. Prediction of Therapeutic Response and Resistance

One of the most promising applications of exosomes lies in their ability to predict treatment response and therapeutic resistance. A recent 2025 study identified a panel of exosomal miRNAs (including miR-130b-3p and miR-454-3p) associated with oxaliplatin resistance in CRC, demonstrating strong diagnostic and predictive performance in both computational and experimental models [39]. These findings suggest that exosomal profiling could enable personalized treatment selection and early identification of non-responders. Moreover, emerging evidence indicates that exosomal signaling pathways contribute directly to therapeutic resistance by transferring resistance-associated molecules between tumor cells, thereby promoting clonal evolution and treatment failure [22].

### 3.11. Clinical Translation and Current Limitations

Despite significant progress, several barriers hinder the clinical implementation of exosome-based biomarkers:

- Lack of standardized isolation and quantification methods [40];
- Inter-study variability in biomarker panels [33,35];
- Limited large-scale prospective validation studies;
- Technical challenges in distinguishing tumor-derived exosomes from normal vesicles [4].

Nevertheless, ongoing technological advances, including high-throughput sequencing and microfluidic-based exosome isolation platforms, are expected to accelerate clinical translation in the near future [32].

Key exosomal biomarkers identified in recent studies are summarized in Table 1.

## 4. Surgical Oncology Implications

From a surgical oncology perspective, exosome-based biomarkers may offer valuable complementary information throughout the perioperative management of colorectal cancer. Following curative-intent resection, one of the greatest clinical challenges is identifying patients who harbor minimal residual disease (MRD) despite apparently complete tumor removal. Although circulating tumor DNA (ctDNA) has emerged as a promising tool for postoperative risk stratification, exosome-derived biomarkers may provide additional biological information by reflecting not only residual tumor burden but also tumor–microenvironment interactions. Rather than replacing ctDNA, exosomal biomarkers may ultimately serve as complementary components of multimodal liquid biopsy strategies.

Another potential application involves postoperative surveillance and prediction of recurrence. Several CRC-associated exosomal miRNAs, including miR-21, miR-92a, and miR-1246, have been associated with aggressive tumor biology, metastatic potential, and poorer clinical outcomes. However, most available evidence originates from retrospective observational studies, and prospective multicenter validation is required before these biomarkers can be incorporated into routine surveillance protocols or influence adjuvant treatment decisions.

Exosome-based monitoring may also become relevant in patients with colorectal liver metastases. Dynamic assessment of circulating exosomal signatures could potentially facilitate earlier detection of metastatic recurrence, evaluation of treatment response after hepatic resection, and longitudinal monitoring during systemic therapy. In the future, integration of exosomal biomarkers with established clinical parameters, imaging findings, and ctDNA analysis may improve clinical decision-support systems for individualized postoperative management. Nevertheless, these applications remain investigational and should currently be considered promising translational concepts rather than established standards of care.

### 4.1. Exosomes and Therapeutic Resistance in Colorectal Cancer

**Figure 2:**
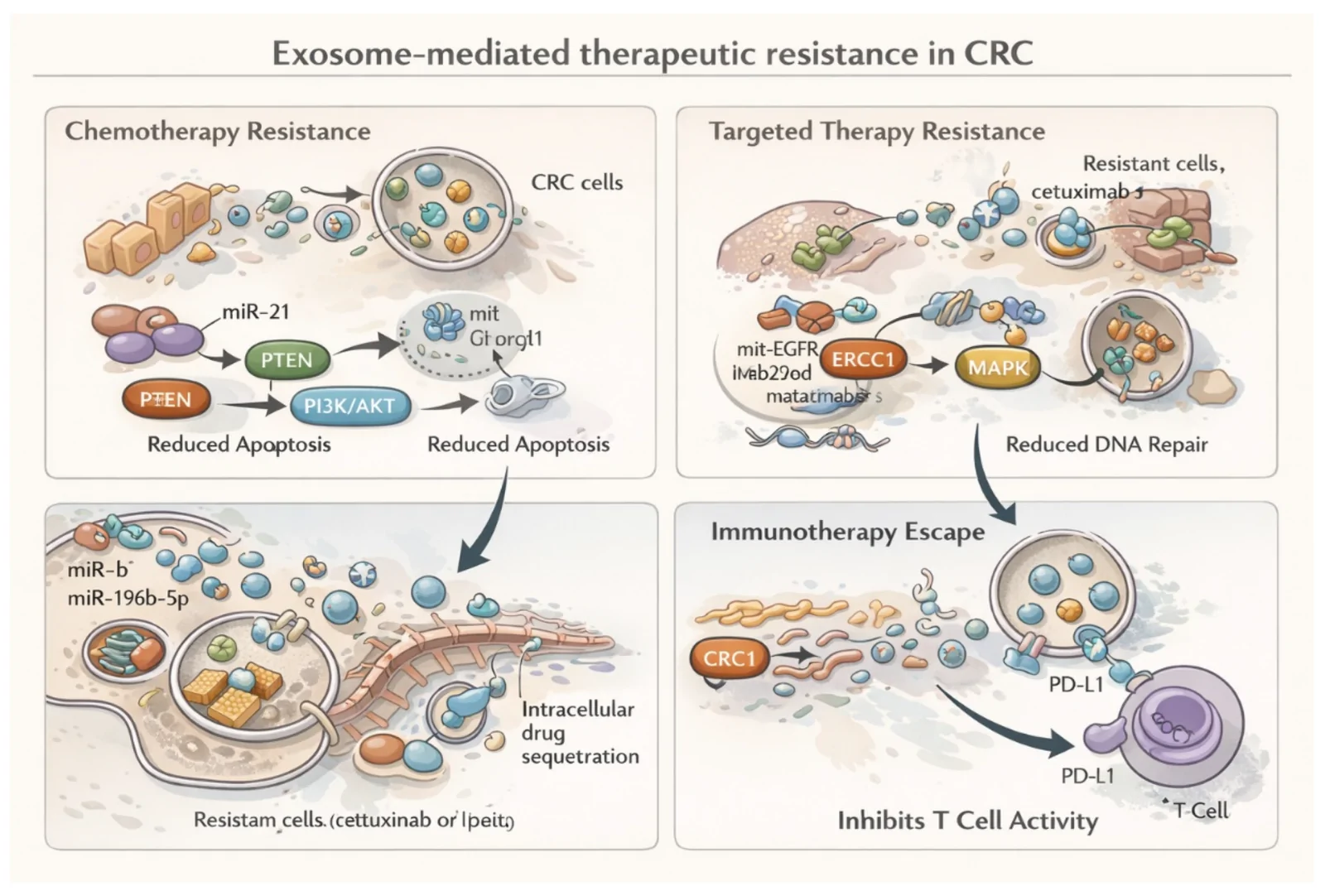
Exosome-mediated therapeutic resistance in CRC: mechanisms of chemotherapy resistance (5-FU, oxaliplatin), targeted therapy resistance (KRAS/EGFR axis), and immunotherapy escape (PD-L1).

Therapeutic resistance remains a major obstacle in the management of colorectal cancer (CRC), significantly limiting the efficacy of chemotherapy, targeted therapies, and immunotherapy. Increasing evidence indicates that exosomes play a pivotal role in mediating both intrinsic and acquired resistance through the horizontal transfer of bioactive molecules that reprogram recipient cells and reshape the tumor microenvironment [4,9]. Key mechanisms of exosome-mediated therapeutic resistance are illustrated in Figure 2.

### 4.2. Exosome-Mediated Chemotherapy Resistance

Resistance to conventional chemotherapeutic agents, particularly 5-fluorouracil (5-FU) and oxaliplatin, represents a critical challenge in CRC treatment. Tumor-derived exosomes contribute to chemoresistance by transferring microRNAs (miRNAs), long non-coding RNAs (lncRNAs), and proteins that regulate apoptosis, drug metabolism, and DNA repair pathways [22].

Exosomal miR-21 has been widely implicated in 5-FU resistance through suppression of tumor suppressor genes such as PTEN and activation of the PI3K/AKT signaling pathway, leading to enhanced cell survival and reduced apoptosis [7]. Similarly, exosomal transfer of miR-196b-5p and miR-208b has been associated with resistance to oxaliplatin by modulating key signaling cascades involved in cell proliferation and survival [41].

Recent studies (2024–2025) have further identified exosomal miRNA signatures predictive of chemotherapy response. Notably, a panel including miR-130b-3p and miR-454-3p has been shown to correlate with oxaliplatin resistance, highlighting the potential of exosomal profiling for treatment stratification [42]. In addition to RNA-mediated mechanisms, exosomes can directly sequester chemotherapeutic agents, reducing intracellular drug accumulation and thereby attenuating cytotoxic efficacy [43].

### 4.3. Targeted Therapy Resistance (Anti-EGFR Axis)

Targeted therapies against the epidermal growth factor receptor (EGFR), such as cetuximab and panitumumab, have improved outcomes in selected CRC patients; however, resistance frequently develops, particularly in the context of KRAS, NRAS, and BRAF mutations. Exosomes have been implicated in mediating resistance to anti-EGFR therapies through the transfer of mutant oncogenic proteins and nucleic acids. For instance, exosomal transmission of mutant KRAS has been shown to confer resistance to EGFR-targeted therapy in previously sensitive cells by reactivating downstream MAPK signaling pathways [44]. Moreover, exosomes derived from resistant CRC cells can induce phenotypic changes in sensitive cells, promoting a resistant phenotype through activation of alternative signaling pathways, including MET and HER2 signaling [45]. These findings suggest that exosomes act as vectors for the horizontal propagation of resistance within heterogeneous tumor cell populations.

### 4.4. Exosomes and Immunotherapy Resistance

Immunotherapy has emerged as a promising treatment modality in CRC, particularly in microsatellite instability-high (MSI-H) tumors. However, the majority of CRC patients exhibit limited responsiveness, highlighting the importance of understanding resistance mechanisms. Exosome-mediated immune modulation plays a central role in immunotherapy resistance. Tumor-derived exosomes carrying programmed death-ligand 1 (PD-L1) can suppress T-cell activation and proliferation, contributing to systemic immune evasion [10]. Importantly, exosomal PD-L1 has been shown to retain functional activity, enabling tumors to exert immunosuppressive effects even at distant sites. Furthermore, exosomes influence the tumor immune microenvironment by promoting the expansion of regulatory T cells (Tregs) and inducing polarization of macrophages toward an immunosuppressive M2 phenotype [31]. These effects collectively impair anti-tumor immune responses and may limit the efficacy of immune checkpoint inhibitors. Emerging evidence also suggests that exosomal cargo may serve as predictive biomarkers for immunotherapy response, although this area remains under active investigation.

### 4.5. Tumor Microenvironment and Stromal-Mediated Resistance

Exosomes facilitate bidirectional communication between tumor cells and stromal components of the tumor microenvironment, including cancer-associated fibroblasts (CAFs), endothelial cells, and immune cells. CAF-derived exosomes have been shown to promote chemoresistance by transferring metabolites, cytokines, and regulatory RNAs that enhance tumor cell survival under therapeutic stress [46]. Conversely, tumor-derived exosomes can reprogram stromal cells to create a protective niche that supports tumor persistence and resistance. Hypoxia, a common feature of the tumor microenvironment, further enhances exosome secretion and alters cargo composition, enriching vesicles with resistance-associated molecules [30]. This adaptive response contributes to treatment failure and disease progression.

### 4.6. Clinical Implications and Future Perspectives

The involvement of exosomes in multiple resistance pathways highlights their potential as both biomarkers and therapeutic targets. Exosomal profiling could enable early identification of resistant phenotypes, facilitating personalized treatment strategies and dynamic treatment monitoring. From a therapeutic standpoint, strategies aimed at inhibiting exosome biogenesis, release, or uptake are currently under investigation. Additionally, engineered exosomes are being explored as delivery vehicles for therapeutic agents designed to overcome resistance mechanisms. However, several challenges remain, including the lack of standardized methodologies, off-target effects, and the complexity of exosome-mediated signaling networks [4,40].

Mechanisms of exosome-mediated therapeutic resistance are outlined in Table 2.

### 4.7. Therapeutic Applications of Exosomes in Colorectal Cancer

**Figure 3:**
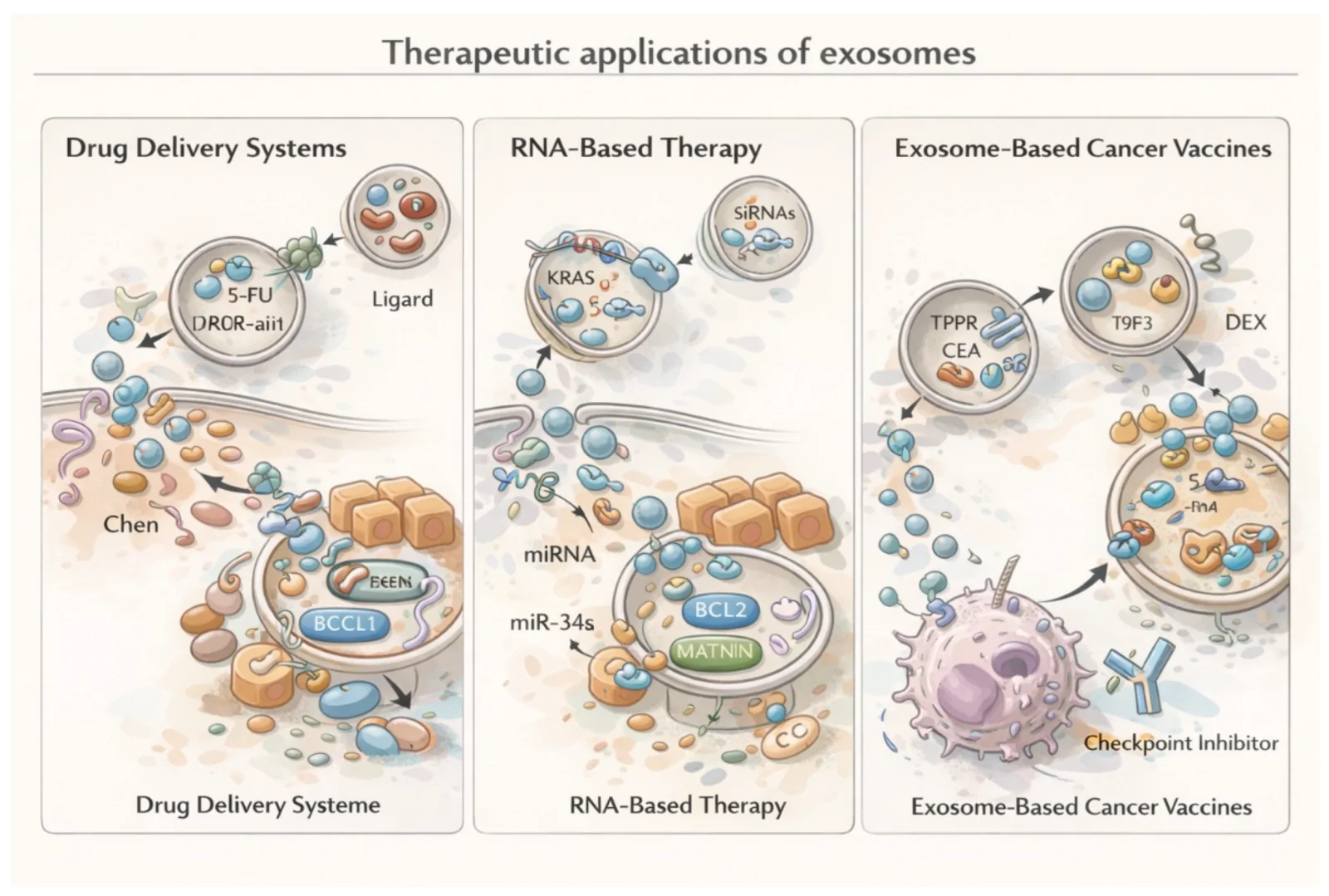
Therapeutic applications of exosomes: drug delivery systems, RNA-based therapy, and exosome-based cancer vaccines.

Beyond their established roles in tumor progression, metastasis, and therapeutic resistance, exosomes have emerged as promising platforms for innovative therapeutic strategies in colorectal cancer (CRC). Their intrinsic biocompatibility, low immunogenicity, and ability to cross biological barriers make them attractive candidates for drug delivery, immunotherapy, and gene-based interventions [4,32]. Emerging therapeutic applications of exosomes in CRC are summarized in Figure 3.

### 4.8. Exosomes as Natural Drug Delivery Vehicles

One of the most extensively investigated therapeutic applications of exosomes is their use as nanoscale drug delivery systems. Due to their lipid bilayer structure and endogenous origin, exosomes exhibit superior stability and reduced immunogenicity compared to synthetic nanoparticles. Preclinical studies have demonstrated that exosomes can be efficiently engineered to encapsulate chemotherapeutic agents such as 5-fluorouracil (5-FU), doxorubicin, and oxaliplatin, thereby enhancing drug stability, tumor targeting, and intracellular uptake while reducing systemic toxicity [13]. Moreover, surface modification strategies (e.g., ligand or antibody conjugation) have enabled tumor-specific targeting, increasing therapeutic precision in CRC models.

### 4.9. RNA-Based Therapeutics and Gene Delivery

Exosomes have also been explored as carriers for RNA-based therapeutics, including small interfering RNAs (siRNAs), microRNAs (miRNAs), and messenger RNAs (mRNAs). Their natural ability to protect nucleic acids from enzymatic degradation makes them particularly suitable for gene therapy applications. Focused experimental studies have shown that exosome-mediated delivery of tumor-suppressive miRNAs, such as miR-34a and miR-16, can inhibit CRC cell proliferation and induce apoptosis by targeting key oncogenic pathways, including BCL2 and Wnt/β-catenin signaling [47]. Similarly, exosome-based siRNA delivery targeting KRAS and PI3K signaling components has demonstrated significant anti-tumor effects in preclinical CRC models [48]. These findings support the concept of exosomes as biological gene transfer vehicles capable of modulating tumor behavior at the genetic and epigenetic levels.

### 4.10. Immunotherapy and Exosome-Based Cancer Vaccines

Exosomes are increasingly being investigated as immunotherapeutic agents, particularly in the context of cancer vaccines. Dendritic cell-derived exosomes (DEXs) can present tumor antigens and activate cytotoxic T lymphocyte (CTL) responses, thereby enhancing anti-tumor immunity. In CRC models, DEX-based vaccines have demonstrated the ability to induce robust immune activation and suppress tumor growth, particularly when combined with immune checkpoint inhibitors [49]. Furthermore, exosomes engineered to express tumor-associated antigens have shown potential in stimulating both innate and adaptive immune responses. However, tumor-derived exosomes can also exert immunosuppressive effects, highlighting the need for careful engineering to shift the balance toward immune activation.

### 4.11. Combination Strategies with Conventional Therapies

Emerging evidence suggests that exosome-based therapeutics may be most effective when used in combination with conventional treatments. For example, exosome-mediated delivery of chemotherapeutic agents has been shown to sensitize resistant CRC cells to 5-FU and oxaliplatin, overcoming drug resistance mechanisms [50]. Additionally, combining exosome-based therapies with immune checkpoint inhibitors (e.g., anti-PD-1/PD-L1) may enhance therapeutic efficacy by simultaneously targeting tumor cells and modulating the immune microenvironment.

### 4.12. Clinical Translation and Ongoing Trials

Despite promising preclinical data, clinical translation of exosome-based therapies remains in its early stages. Several challenges persist, including large-scale production, purification standardization, cargo loading efficiency, and regulatory considerations. Nevertheless, early-phase clinical studies investigating exosome-based drug delivery systems and dendritic cell-derived exosome vaccines are currently ongoing, reflecting growing interest in their translational potential [51].

Emerging therapeutic applications of exosomes are presented in Table 3.

### 4.13. Challenges and Future Directions

Key obstacles limiting clinical implementation include:

- Lack of standardized exosome isolation and engineering protocols;
- Heterogeneity of exosomal populations;
- Scalability of production for clinical-grade applications;
- Safety concerns regarding off-target effects and biodistribution.

Future advances in nanotechnology, synthetic biology, and bioengineering are expected to overcome these barriers, paving the way for precision exosome-based therapeutics in CRC [4,40].

## 5. Critical Synthesis: Current Controversies, Evidence Gaps, and Translational Limitations

Although exosomes have generated considerable interest in colorectal cancer research, the current evidence remains heterogeneous and largely preclinical. Most studies demonstrate mechanistic associations between exosomal cargo and tumor progression, metastasis, immune escape, or treatment resistance; however, causal relationships in clinically relevant human cohorts remain insufficiently validated. Therefore, exosomes should currently be viewed as promising translational candidates rather than established clinical tools.

A major controversy concerns the specificity of tumor-derived exosomes. Circulating extracellular vesicles originate from multiple cell types, including immune cells, platelets, stromal cells, and normal epithelial tissues. As a result, distinguishing cancer-specific exosomal signatures from background vesicle populations remains technically challenging. This limitation is particularly important for biomarker studies, where reported miRNA or circRNA signatures often vary across cohorts, platforms, and isolation methods.

Another unresolved issue is methodological standardization. Differences in exosome isolation, purification, quantification, and cargo analysis may partly explain the inconsistent findings reported across studies. Ultracentrifugation, size-exclusion chromatography, precipitation-based kits, and microfluidic platforms can yield vesicle populations with different purity and molecular profiles. Consequently, many proposed biomarkers require independent validation using standardized protocols before they can be incorporated into clinical practice.

The diagnostic and prognostic utility of exosomal biomarkers also remains incompletely confirmed. Although several exosomal miRNAs and non-coding RNAs have shown promising performance for CRC detection, metastasis prediction, or therapy-response monitoring, most studies are retrospective, single-center, or based on limited sample sizes. Large prospective studies comparing exosome-based assays with established modalities such as colonoscopy, carcinoembryonic antigen, ctDNA, and imaging are still needed.

Therapeutic applications of exosomes are even less mature. Exosome-based drug delivery, RNA therapeutics, and cancer vaccines have shown encouraging results in experimental models, but their clinical translation remains limited by challenges related to scalable production, cargo loading efficiency, biodistribution, off-target effects, immunogenicity, and regulatory approval. Thus, although exosomes represent attractive therapeutic platforms, most applications remain preclinical and should not yet be interpreted as ready for routine clinical use.

Finally, the biological role of exosomes may be context-dependent. The same exosomal molecule may have different effects depending on tumor stage, molecular subtype, microenvironmental conditions, and treatment exposure. This complexity highlights the need for integrated multi-omics approaches, functional validation, and longitudinal clinical sampling. Future research should prioritize standardized methodology, multicenter validation, clinically annotated cohorts, and direct comparison with existing biomarkers and therapeutic strategies.

## 6. Current Clinical Readiness of Exosome-Based Applications in Colorectal Cancer

Although exosome-based technologies have demonstrated substantial promise in colorectal cancer research, their level of clinical maturity varies considerably depending on the intended application. To avoid overinterpretation of the current evidence, it is important to distinguish between preclinical discoveries, early translational studies, retrospective biomarker investigations, prospective clinical validation, and routine clinical implementation.

At present, most mechanistic knowledge regarding exosome biology originates from in vitro experiments and animal models, which have established important roles for exosomes in tumor progression, liver metastasis, immune modulation, and therapeutic resistance. These findings provide strong biological rationale but do not, by themselves, establish clinical utility.

The majority of proposed exosomal biomarkers—including exosomal miRNAs, circRNAs, lncRNAs, proteins, and extracellular vesicle signatures—have been evaluated primarily in retrospective observational studies or relatively small patient cohorts. Although several biomarkers have demonstrated encouraging diagnostic or prognostic performance, independent multicenter validation remains limited, and few studies have directly compared these markers with established clinical standards such as colonoscopy, carcinoembryonic antigen (CEA), circulating tumor DNA (ctDNA), or guideline-recommended surveillance strategies.

Similarly, therapeutic applications of engineered exosomes, including drug delivery systems, RNA therapeutics, and cancer vaccines, remain largely preclinical. Early translational studies have demonstrated feasibility, but robust clinical evidence supporting efficacy, long-term safety, optimal manufacturing procedures, regulatory approval, and cost-effectiveness is currently lacking.

Consequently, no exosome-based biomarker or therapeutic strategy currently constitutes a standard-of-care approach for colorectal cancer management. Before routine clinical implementation can be recommended, large prospective multicenter studies, standardized isolation and characterization protocols, analytical validation, clinical qualification, and demonstration of clinical utility will be required. Therefore, exosome-based technologies should presently be regarded as investigational tools with considerable translational potential rather than established components of routine colorectal cancer care.

## 7. Current Challenges and Controversies

Despite encouraging progress, several important challenges continue to limit the clinical translation of exosome-based biomarkers in colorectal cancer. Considerable variability exists among studies due to differences in patient populations, sample processing, exosome isolation methods, and analytical platforms, resulting in inconsistent biomarker performance and limited reproducibility [4,40]. Furthermore, distinguishing tumor-derived exosomes from the heterogeneous extracellular vesicle population remains technically challenging, while most proposed biomarkers have been evaluated only in retrospective or small single-center studies [32,33,35]. Another unresolved issue is whether exosome-based biomarkers provide incremental clinical value over established approaches such as circulating tumor DNA (ctDNA) and conventional clinicopathological parameters. Therefore, standardized methodologies and large prospective multicenter studies are essential before exosome-based biomarkers can be incorporated into routine colorectal cancer management [40,51].

## 8. Conclusions and Future Perspectives

**Figure 4:**
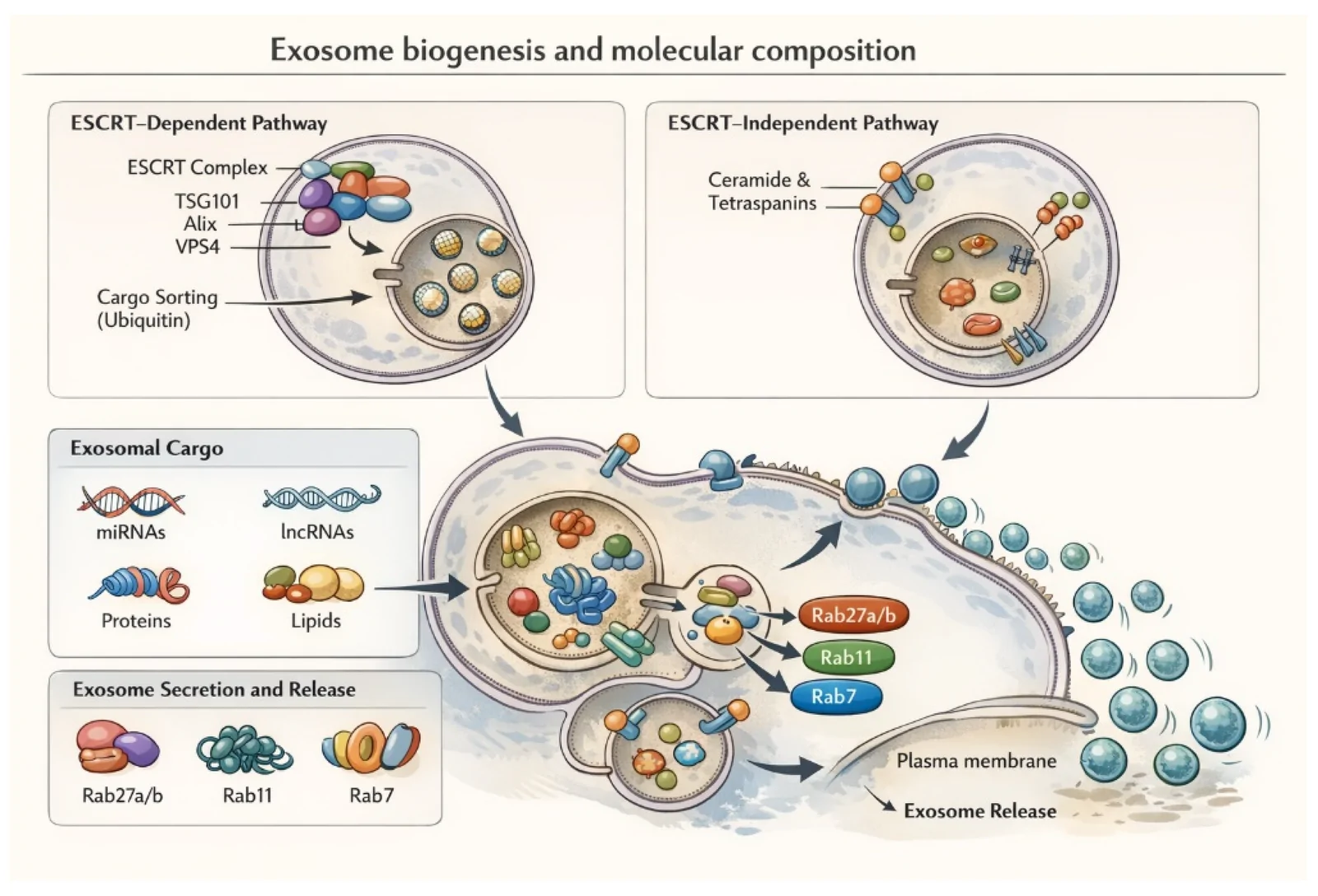
Exosome biogenesis and molecular composition. Schematic illustration of ESCRT-dependent and independent exosome formation pathways, cargo sorting mechanisms, and secretion via Rab GTPases.

Exosomes have emerged as pivotal regulators of colorectal cancer (CRC) biology, evolving from passive cellular byproducts to active mediators of intercellular communication. Growing evidence indicates that they play critical roles in tumor initiation, progression, metastatic dissemination, immune evasion, and therapeutic resistance by facilitating the horizontal transfer of bioactive cargo, including proteins, nucleic acids, and lipids [4,9]. The biogenesis and secretion pathways of exosomes are illustrated in Figure 4.

From a diagnostic standpoint, exosomes represent a highly promising platform for liquid biopsy applications, offering superior molecular stability and the ability to reflect real-time tumor dynamics. Recent advances in multi-omics profiling have further expanded their utility as biomarkers for early detection, prognostic stratification, and prediction of therapeutic response. In parallel, their role in mediating chemoresistance, targeted therapy failure, and immunotherapy escape highlights their clinical relevance as dynamic regulators of treatment outcomes [32].

Therapeutically, exosomes have transitioned from being solely biomarkers to versatile bio-nanocarriers with significant translational potential. Their application in drug delivery, RNA-based therapeutics, and cancer immunotherapy underscores a paradigm shift toward exosome-engineered precision medicine. However, despite encouraging preclinical data, clinical translation remains limited due to challenges in large-scale production, purification standardization, cargo heterogeneity, and regulatory constraints [40].

Future research should focus on addressing these limitations through the development of standardized isolation protocols, advanced bioengineering strategies, and robust clinical validation studies. Integration of artificial intelligence-driven multi-omics analyses may further enhance the predictive power of exosome-based biomarkers and facilitate patient-specific therapeutic strategies.

Importantly, from a surgical oncology perspective, exosome biology may provide novel opportunities for perioperative risk stratification, early detection of minimal residual disease, and prediction of metastatic recurrence following curative resection. The ability to detect tumor-derived exosomal signatures in the postoperative period could significantly refine follow-up strategies and guide adjuvant treatment decisions, particularly in high-risk CRC patients.

In conclusion, exosomes represent a fundamental link between tumor biology and clinical oncology. Their dual role as both mediators of disease progression and tools for diagnosis and therapy positions them at the forefront of translational cancer research. Continued interdisciplinary efforts are essential to fully harness their potential and integrate exosome-based technologies into routine clinical practice for colorectal cancer management.

In the era of precision oncology, exosomes may not only serve as biomarkers but also as dynamic regulators of disease trajectory, potentially redefining how colorectal cancer is diagnosed, monitored, and treated.

The added value of the present review lies not only in summarizing recent advances but also in critically evaluating the translational readiness of exosome-based applications in colorectal cancer. Rather than providing a purely descriptive overview, we emphasize unresolved methodological challenges, limitations of current biomarker validation, perioperative surveillance opportunities, and the potential integration of exosome-based technologies into surgical oncology practice. We believe this clinically oriented perspective differentiates the present review from previously published articles and highlights priorities for future translational research.

## Figures and Tables

**Table 1:** Diagnostic and prognostic exosomal biomarkers in CRC (2023–2025).

Biomarker	Molecule Type	Clinical Role	Mechanism	Clinical Relevance	Reported Diagnostic Performance (Where Available)
miR-99b-5p	miRNA	Diagnostic	Regulates tumor-related signaling pathways	Early CRC detection (high sensitivity/specificity)	High diagnostic accuracy reported in systematic reviews; quantitative values vary across individual studies and were not uniformly reported [34,35]
miR-205-5p	miRNA	Diagnostic	Modulates epithelial cell proliferation	Elevated in CRC patients vs. controls	Diagnostic potential reported; however, no quantitative diagnostic metrics are available in the cited references.
circCOG2	circRNA	Prognostic	miRNA sponging via the miR-1305/TGF-β2/SMAD3 pathway, promotes tumor growth	Associated with tumor progression, invasion, and poor prognosis	Clinical and mechanistic evidence supports its prognostic significance; however, diagnostic performance metrics (AUC, sensitivity, specificity) were not reported in the original study [36]
miR-1246	miRNA	Prognostic/Metastasis	Activates hepatic stellate cells	Predicts liver metastasis	Strong prognostic evidence for liver metastasis; diagnostic sensitivity/specificity not reported [21]
miR-320d	miRNA	Prognostic	Activates JAK/STAT signaling	Correlates with aggressive tumor phenotype	Mechanistic and prognostic evidence reported; diagnostic performance metrics were not evaluated [37]
miR-21	miRNA	Diagnostic/Prognostic	Targets PTEN, activates PI3K/AKT	Associated with poor prognosis	Frequently reported as a diagnostic biomarker; quantitative performance varies among studies and systematic reviews [19,34,35]
miR-92a	miRNA	Diagnostic	Regulates angiogenesis-related pathways	Non-invasive CRC biomarker	Included among promising circulating diagnostic miRNAs; pooled diagnostic performance available in systematic reviews but not reported as an individual exosomal biomarker [34,35]
miR-130b-3p	miRNA	Predictive	Modulates apoptosis and drug resistance pathways	Predicts oxaliplatin resistance	Identified as part of a predictive exosomal miRNA signature for oxaliplatin resistance; no independent diagnostic sensitivity/specificity reported [39]
miR-454-3p	miRNA	Predictive	Enhances survival signaling	Associated with chemotherapy resistance	Identified as part of a predictive exosomal miRNA signature; no independent diagnostic performance reported [39]

**Table 2:** Exosome-mediated therapeutic resistance mechanisms in CRC.

Drug Type	Exosomal Molecule	Target Pathway	Mechanism	Clinical Effect
5-FU	miR-21	PI3K/AKT	PTEN suppression anti-apoptosis	Chemotherapy resistance
Oxaliplatin	miR-196b-5p	MAPK/Survival pathways	Enhances proliferation	Drug resistance
Oxaliplatin	miR-208b	Cell cycle regulation	Promotes survival	Reduced drug response
Chemotherapy (general)	miR-130b-3p/miR-454-3p	Apoptosis pathways	Alters drug sensitivity	Predictive biomarker
Anti-EGFR therapy	Mutant KRAS (exosomal)	MAPK signaling	Reactivates downstream signaling	Resistance to cetuximab
Anti-EGFR therapy	HER2/MET signals	Alternative RTK pathways	Bypass signaling activation	Targeted therapy failure
Immunotherapy	PD-L1 (exosomal)	Immune checkpoint pathway	T-cell inhibition	Immune escape
Multiple therapies	lncRNAs/proteins	Various	Horizontal transfer of resistance traits	Tumor heterogeneity & resistance
Chemotherapy	Drug sequestration	Intracellular drug levels	Drug efflux via exosomes	Reduced cytotoxicity

**Table 3:** Therapeutic applications of exosomes in CRC.

Application Type	Strategy	Example	Mechanism	Clinical Potential
Drug Delivery	Chemotherapy loading	5-FU, Oxaliplatin	Targeted delivery via lipid vesicles	Increased efficacy, reduced toxicity
Drug Delivery	Surface engineering	Ligand-modified exosomes	Tumor-specific targeting	Precision oncology
Gene Therapy	miRNA delivery	miR-34a, miR-16	Tumor suppressor activation	Inhibits proliferation
Gene Therapy	siRNA delivery	KRAS-targeting siRNA	Gene silencing	Anti-tumor effect
Immunotherapy	Dendritic cell exosomes	Tumor antigen presentation	Activates T cells	Cancer vaccine strategy
Immunotherapy	Engineered exosomes	PD-1/PD-L1 modulation	Immune activation	Enhances checkpoint therapy
Combination Therapy	Chemo + exosomes	Drug sensitization	Overcomes resistance	Improved treatment response
Precision Medicine	Personalized exosomes	Patient-derived vesicles	Tailored therapy	Future clinical application

## Data Availability

No new data were created or analyzed in this study.
